# Constructions of Unextendible Maximally Entangled Bases in ${{\mathbb{C}}}^{d}\otimes {{\mathbb{C}}}^{d^{\prime} }$

**DOI:** 10.1038/s41598-018-21561-0

**Published:** 2018-02-16

**Authors:** Gui-Jun Zhang, Yuan-Hong Tao, Yi-Fan Han, Xin-Lei Yong, Shao-Ming Fei

**Affiliations:** 1 Department of Mathematics College of Sciences, Yanbian University, Yanji, 133002 China; 2 School of Mathematics Sciences, Capital Normal University, Beijing, 100048 China; 3 Max-Planck-Institute for Mathematics in the Science, Leipzig, 04103 Germany

## Abstract

We study unextendible maximally entangled bases (UMEBs) in ${{\mathbb{C}}}^{d}\otimes {{\mathbb{C}}}^{d^{\prime} }$ (*d* < *d*′). An operational method to construct UMEBs containing *d*(*d*′ − 1) maximally entangled vectors is established, and two UMEBs in ${{\mathbb{C}}}^{5}\otimes {{\mathbb{C}}}^{6}$ and ${{\mathbb{C}}}^{5}\otimes {{\mathbb{C}}}^{12}$ are given as examples. Furthermore, a systematic way of constructing UMEBs containing *d*(*d*′ − *r*) maximally entangled vectors in ${{\mathbb{C}}}^{d}\otimes {{\mathbb{C}}}^{d^{\prime} }$ is presented for *r* = 1, 2, …, *d* − 1. Correspondingly, two UMEBs in ${{\mathbb{C}}}^{3}\otimes {{\mathbb{C}}}^{10}$ are obtained.

## Introduction

Quantum entanglement lies in the heart of the quantum information processing. It plays important roles in many fields such as quantum teleportation, quantum coding, quantum key distribution protocol, quantum non-locality^1–4^. Quantum teleportation, which can be used for distributed quantum learning^5^ and even in organisms^6^, is a essential element in quantum information processing. Maximally entangled states attract much attention due to their importance in ensuring the highest fidelity and efficiency in quantum teleportation^7^. A pure state |*ψ*〉 is said to be a *d* ⊗ *d*′ (*d* < *d*′) maximally entangled state if and only if for an arbitrary given orthonormal basis {|*i*_*A*_〉} of subsystem A, there exists an orthonormal basis {|*i*_*B*_〉} of subsystem B such that |*ψ*〉 can be written as $|\psi \rangle =\frac{1}{\sqrt{d}}{\sum }_{i=0}^{d-1}|{i}_{A}\rangle \otimes |{i}_{B}\rangle$^8^.

Nonlocality is a very useful concept in quantum mechanics^9–13^ and plays an important role in Van der Waals interaction in transformation optics^14^. It is tightly related to entanglement. While, it is proven that the unextendible product bases (UPBs) reveal some nolocality without entanglement^15,16^. The UPB is a set of incomplete orthogonal product states in bipartite quantum system ${{\mathbb{C}}}^{d}\otimes {{\mathbb{C}}}^{d^{\prime} }$ consisting of fewer than *dd*′ vectors which have no additional product states are orthogonal to each element of the set^17^.

A UPB in ${{\mathbb{C}}}^{3}\otimes {{\mathbb{C}}}^{3}$ with 5 pure states is as follows^17^:

$$|{{\varphi }}_{0}\rangle =\frac{1}{\sqrt{2}}\mathrm{|0}\rangle \otimes \mathrm{(|0}\rangle -\mathrm{|1}\rangle ),\quad |{{\varphi }}_{1}\rangle =\frac{1}{\sqrt{2}}\mathrm{(|0}\rangle -\mathrm{|1}\rangle )\otimes \mathrm{|2}\rangle ,$$

$$|{{\varphi }}_{2}\rangle =\frac{1}{\sqrt{2}}\mathrm{|2}\rangle \otimes \mathrm{(|1}\rangle -\mathrm{|2}\rangle ),\quad |{{\varphi }}_{3}\rangle =\frac{1}{\sqrt{2}}\mathrm{(|1}\rangle -\mathrm{|2}\rangle )\otimes \mathrm{|0}\rangle ,$$

$$|{{\varphi }}_{4}\rangle =\frac{1}{3}\mathrm{(|0}\rangle +\mathrm{|1}\rangle +\mathrm{|2}\rangle )\otimes \mathrm{(|0}\rangle +\mathrm{|1}\rangle +\mathrm{|2}\rangle \mathrm{).}$$

Obviously, they are all product states.

There exist a nonzero pure state |*ψ*〉, which is orthogonal to |*ϕ*_*i*_〉(*i* = 0, 1, 2, 3, 4). If |*ψ*〉 is a product state, it can be expressed as |*ψ*〉 = (*a*|0〉 + *b*|1〉 + *c*|2〉) ⊗ (*a*′|0〉 + *b*′|1〉 + *c*′|2〉), where *a*^2^ + *b*^2^ + *c*^2^ = *a*′^2^ + *b*′^2^ + *c*′^2^ = 1. No loss of generalization, we assume that *a*, *a*′ ≠ 0. From |*ψ*〉 is orthogonal to |*ϕ*_0_〉, we have *b*′ = *a*′ ≠ 0. Due to |*ψ*〉 is orthogonal to |*ϕ*_4_〉, we can conclude that *c*′ ≠ 0. Because of that |*ψ*〉 is orthogonal to |*ϕ*_1_〉, we have *b* = *a* ≠ 0. Owing to that |*ψ*〉 is orthogonal to |*ϕ*_3_〉, we obtain that *c* = *b* ≠ 0. And |*ψ*〉 is orthogonal to |*ϕ*_2_〉, we get that *c*′ = *b*′ ≠ 0. That is to say, *a* = *b* = *c* = $\frac{1}{\sqrt{3}}$ and *a*′ = *b*′ = *c*′ = $\frac{1}{\sqrt{3}}$. Now |*ψ*〉 is equal to |*ϕ*_4_〉, instead of being orthogonal to |*ϕ*_4_〉. Therefore, |*ψ*〉 can not be a product state. That’s why the set $\{|{{\varphi }}_{i}\rangle {\}}_{i=0}^{4}$ is described by ‘unextendible’ and $\{|{{\varphi }}_{i}\rangle {\}}_{i=0}^{4}$ is a UPB in ${{\mathbb{C}}}^{3}\otimes {{\mathbb{C}}}^{3}$.

Bravyi and Smolin^18^ generalized the notion of UPB to unextendible maximally entangled bases (UMEB): a set of incomplete orthogonal maximally entangled states in bipartite quantum system ${{\mathbb{C}}}^{d}\otimes {{\mathbb{C}}}^{d^{\prime} }$ consisting of fewer than *dd*^′^ vectors which have no additional maximally entangled vectors orthogonal to all of them. They state that UMEBs can be used to construct examples of states for which 1-copy entanglement of assistance (EoA) is strictly smaller than the asymptotic EoA and find quantum channels that are unital but not convex mixtures of unitary operations^18^.

Let {|0〉, |1〉} be a orthogonal base of ${{\mathbb{C}}}^{2}$. And {|0′〉, |1′〉, |2′〉} be a orthogonal base of ${{\mathbb{C}}}^{3}$. Then, we present a UMEB in ${{\mathbb{C}}}^{2}\otimes {{\mathbb{C}}}^{3}$^19^:

$$|{{\varphi }}_{\mathrm{1,2}}\rangle =\frac{1}{\sqrt{2}}\mathrm{(|0}\rangle \otimes |0^{\prime} \rangle \pm \mathrm{|1}\rangle \otimes |1^{\prime} \rangle ),$$

$$|{{\varphi }}_{\mathrm{3,4}}\rangle =\frac{1}{\sqrt{2}}\mathrm{(|0}\rangle \otimes |1^{\prime} \rangle \pm \mathrm{|1}\rangle \otimes |0^{\prime} \rangle \mathrm{).}$$

Obviously, they are all maximally entangled states.

If a nonzero pure state |*ψ*〉 is orthogonal to |*ϕ*_*i*_〉(*i* = 1, 2, 3, 4), it’s sure that |*ψ*〉 = (*a*|0〉 + *b*|1〉) ⊗ |2′〉, where *a*^2^ + *b*^2^ = 1. In other words, |*ψ*〉 must be a product state, rather than maximally entangled states. Hence $\{|{{\varphi }}_{i}\rangle {\}}_{i=0}^{4}$ is a UMEB in ${{\mathbb{C}}}^{2}\otimes {{\mathbb{C}}}^{3}$.

The number of the vectors in a UMEB is less than the dimension of the bipartite system space. Therefore a UMEB in ${{\mathbb{C}}}^{d}\otimes {{\mathbb{C}}}^{d^{\prime} }$ containing *n* maximally entangled vectors is usually expressed as a *n*-number UMBE, when *n* is smaller than *dd*^′^. Chen and Fei^19^ provided a way to construct *d*^2^-member UMEBs in ${{\mathbb{C}}}^{d}\otimes {{\mathbb{C}}}^{d^{\prime} }$ $(\frac{d^{\prime} }{2} < d < d^{\prime} )$. Later, Nan *et al*.^20^ and Li *et al*.^21^ constructed two sets of UMEBs in ${{\mathbb{C}}}^{d}\otimes {{\mathbb{C}}}^{d^{\prime} }$ (*d* < *d*′) independently. Wang *et al*.^22^ put forward a method of constructing UMEBs in ${{\mathbb{C}}}^{qd}\otimes {{\mathbb{C}}}^{qd}$ from that in ${{\mathbb{C}}}^{d}\otimes {{\mathbb{C}}}^{d}$, and gave a 30-member UMEB in ${{\mathbb{C}}}^{6}\otimes {{\mathbb{C}}}^{6}$. They proved that there exist UMEBs in ${{\mathbb{C}}}^{d}\otimes {{\mathbb{C}}}^{d}$ except for *d* = *p* or 2*p*, where *p* is a prime and *p* = 3 mod 4. They also presented a 23-member UMEB in ${{\mathbb{C}}}^{5}\otimes {{\mathbb{C}}}^{5}$ and a 45-member UMEB in ${{\mathbb{C}}}^{7}\otimes {{\mathbb{C}}}^{7}$^23^. Then Guo^24–26^ proposed a scenario of constructing UMEBs via the space decomposition, which improves the previous work about UMEBs.

In this paper, we give two methods of constructing UMEBs in ${{\mathbb{C}}}^{d}\otimes {{\mathbb{C}}}^{d^{\prime} }(d\le d^{\prime} )$. In Sec. 2 we first recall some basic notions and lemmas about UMEB and space decomposition. In Sec. 3 we give an operational method to construct *d*(*d*′ − 1)-number UMEB and then present explicit constructions of UMEBs in ${{\mathbb{C}}}^{5}\otimes {{\mathbb{C}}}^{6}$ and ${{\mathbb{C}}}^{5}\otimes {{\mathbb{C}}}^{12}$. In Sec. 4 we present an approach of systematically constructing *d*(*d*′ − *r*)-member UMEBs in ${{\mathbb{C}}}^{d}\otimes {\mathbb{C}}d^{\prime}$ for *r* = 1, 2, …, *d* − 1, and give two examples in ${{\mathbb{C}}}^{3}\otimes {{\mathbb{C}}}^{10}$. We summarize in Sec. 5.

## Preliminaries

Throughout this paper, we assume that *d* < *d*^′^. Let us first recall some basic notions and lemmas^18,19,24^. Let {|*k*〉} and $\{|\ell ^{\prime} \rangle \}$ be the standard computational bases of ${{\mathbb{C}}}^{d}$ and ${{\mathbb{C}}}^{d^{\prime} }$, respectively, and $\{|{{\varphi }}_{i}\rangle {\}}_{i=1}^{dd^{\prime} }$ an orthonormal basis of ${{\mathbb{C}}}^{d}\otimes {{\mathbb{C}}}^{d^{\prime} }$. Let *M*_*d*×*d*′_ be the Hilbert space of all *d* × *d*′ complex matrices equipped with the inner product defined by 〈*A*|*B*〉 = *Tr*(*A*^†^*B*) for any *A*, *B* ∈ *M*_*d*×*d*′_. If ${\{{A}_{i}\}}_{i=1}^{dd^{\prime} }$ constitutes a Hilbert-Schmidt basis of *M*_*d*×*d*′_, where 〈*A*_*i*_|*A*_*j*_〉 = *dδ*_*ij*_, then there is a one-to-one correspondence between {|*ϕ*_*i*_〉} and {*A*_*i*_} as follows^25,26^:

$$|{{\varphi }}_{i}\rangle =\sum _{k,\ell }{a}_{kl}^{(i)}|k\rangle |\ell ^{\prime} \rangle \in {{\mathbb{C}}}^{d}\otimes {{\mathbb{C}}}^{d^{\prime} }\,\,\iff \,\,{A}_{i}=[\sqrt{d}{a}_{k\ell }^{(i)}]\in {M}_{d\times d^{\prime} },$$

1 $$Sr(|{{\varphi }}_{i}\rangle )=rank({A}_{i}),\,\,\,\,\langle {{\varphi }}_{i}|{{\varphi }}_{j}\rangle =\frac{1}{d}Tr({A}_{i}^{\dagger }{A}_{j}),$$

where *Sr*(|*ϕ*_*i*_〉) denotes the Schmidt number of |*ϕ*_*i*_〉. Obviously, |*ϕ*_*i*_〉 is a maximally entangled pure state in *C*^*d*^ ⊗ *C*^*d*′^ iff (*d*)^1/2^*A*_*i*_ is a *d* × *d*′ singular-value-1 matrix (a matrix whose singular values all equal to 1).

A basis $\{|{{\varphi }}_{i}\rangle {\}}_{i=1}^{dd^{\prime} }$ constituted by maximally entangled states in *C*^*d*^ ⊗ *C*^*d*′^ is called a maximally entangled basis (MEB) of *C*^*d*^ ⊗ *C*^*d*′^. A set of pure states $\{|{{\varphi }}_{i}\rangle {\}}_{i=1}^{n}\in {C}^{d}\otimes {C}^{d^{\prime} }$ with the following conditions is called an unextendible maximally entangled basis (UMEB)^18,19^:

(i) |*ϕ*_*i*_〉, *i* = 1, 2, 3 ... *n* are all maximally entangled states.
(ii) $$\langle {\varphi }_{i}|{\varphi }_{j}\rangle ={\delta }_{ij},\,i,j=1,\,2,\,3,\ldots ,\,n$$.
(iii) *n* < *dd*′, and if a pure state |*ψ*〉 satisfies that 〈*ϕ*_*i*_|*ψ*〉 = 0, *i* = 1, 2, 3... *n*, then |*ψ*〉 can not be maximally entangled.

A Hilbert-Schmidt basis ${\{{A}_{i}\}}_{i=1}^{dd^{\prime} }$ constituted by single-value-1 matrices in *M*_*d*×*d*′_ is called single-value-1 Hilbert-Schmidt basis (SV1B) of *M*_*d*×*d*′_. A set of *d* × *d*′ matrices ${\{{A}_{i}\}}_{i=1}^{n}$ with the following conditions is called unextendible singular-value-1 Hilbert-Schmidt basis (USV1B) of *M*_*d*×*d*′_^24^:

(i) *A*_*i*_, *i* = 1, 2, 3 ... *n* are all single-value-1 matrices.
(ii) $$Tr({A}_{i}^{\dagger }{A}_{j})=d{\delta }_{ij}$$, *i*, *j* = 1, 2, 3... *n*.
(iii) *n* < *dd*′, and if a matrix *X* satisfies that *Tr*(*X*^†^*A*_*i*_) = 0, *i* = 1, 2, 3... *n*, then *X* can not be a single-value-1 matrix.

It is obvious that ${\{{A}_{i}\}}_{i\mathrm{=1}}^{dd^{\prime} }$ is an SV1B of *M*_*d*×*d*′_ iff $\{|{{\varphi }}_{i}\rangle {\}}_{i=1}^{dd^{\prime} }$ is a MEB of *C*^*d*^ ⊗ *C*^*d*′^, and ${\{{A}_{i}\}}_{i\mathrm{=1}}^{n}$ is a USV1B of *M*_*d* × *d*′_ iff $\{|{{\varphi }}_{i}\rangle {\}}_{i\mathrm{=1}}^{n}$ is a UMEB of *C*^*d*^ ⊗ *C*^*d*′^. Therefore, for convenience, we may just call an SV1B ${\{{A}_{i}\}}_{i\mathrm{=1}}^{dd^{\prime} }$ of *M*_*d* × *d*′_ an MEB $\{|{{\varphi }}_{i}\rangle {\}}_{i\mathrm{=1}}^{dd^{\prime} }$ of ${{\mathbb{C}}}^{d}\otimes {{\mathbb{C}}}^{d^{\prime} }$, and call a USV1B ${\{{A}_{i}\}}_{i\mathrm{=1}}^{n}$ of *M*_*d*×*d*′_ a UMEB $\{|{{\varphi }}_{i}\rangle {\}}_{i\mathrm{=1}}^{n}$ of ${{\mathbb{C}}}^{d}\otimes {{\mathbb{C}}}^{d^{\prime} }$.

In deriving our main results, we need the following lemma in ref.^24^.

### **Lemma 1**.

Let ${M}_{d\times d^{\prime} }={M}_{1}\oplus {M}_{1}^{\perp }$. If {|*ϕ*_*i*_〉} is a MEB in *M*_1_ and {|*ψ*_*i*_〉} is a UMEB in ${M}_{1}^{\perp }$, then {|*ϕ*_*i*_〉}∪{|*ψ*_*i*_〉} is a UMEB in *M*^24^. If {|*ϕ*_*i*_〉} is a MEB in *M*_1_ and ${M}_{1}^{\perp }$ contains no single-value-1 matrix (maximally entangled state), then {|*ϕ*_*i*_〉} is a UMEB in *M*.

## *d*(*d*′ − 1)-member UMEBs in ${{\mathbb{C}}}^{d}\otimes {{\mathbb{C}}}^{d^{\prime} }$

In this section, we will establish a flexible method to construct *d*(*d*′ − 1)-member UMEBs in ${{\mathbb{C}}}^{d}\otimes {{\mathbb{C}}}^{d^{\prime} }$.

### **Theorem 1**.

Let *M*_*d*×*d*′_ be the Hilbert space of all *d* × *d*′ complex matrices. If *V* is a subspace of *M*_*d*×*d*′_ such that each matrix in *V* is a *d* × *d*′ matrix ignoring *d* entries which occupy different rows and *N* columns with *N* < *d*, then there exists a *d*(*d*′ − 1)-member MEB in *V*, as well as a *d*(*d*′ − 1)-member UMEB in *M*_*d*×*d*′_.

### **Proof**.

Without loss of generality, we can always assume the ignored *d* entries in *V* only occupy the former *N* columns. Let *b*_*i*_, *i* = 0, 1, ..., *d* − 1, denote the column number of the ignored element in the *i*-th row. Obviously, *b*_*i*+1_ − *b*_*i*_ = 0 or 1.

Denote

2 $$C(k,\,l)=\{\begin{array}{cc}1, & l={b}_{k};\\ 0, & otherwise.\end{array}$$

We can construct *d*(*d*′ − 1) pure states in ${{\mathbb{C}}}^{d}\otimes {{\mathbb{C}}}^{d^{\prime} }$ as follows,

3 $$|\varphi {^{\prime} }_{j,n}\rangle =\frac{1}{\sqrt{d}}\sum _{m=0}^{d-1}{\omega }_{d}^{nm}|m\rangle |{t}_{mj}\rangle ,\,j=0,\,1,\ldots ,\,d^{\prime} -2;\,n=0,\,1,\ldots ,d-1,$$

where $${\omega }_{d}={e}^{\frac{2\pi \sqrt{-1}}{d}}$$, and

4 $${t}_{mj}=\{\begin{array}{cc}j+1, & m=0;\\ {t}_{m-1,j}+1{\oplus }_{d^{\prime} }C(m,{t}_{m-1,j}+1), & m=1,\,2,\,\ldots ,\,d-1,\end{array}$$

*p* ⊕ _*d*′_*m* denotes (*p* + *m*) mod *d*′.

Next, we prove that all the states in (3) constitute an MEB in *V*.

(i) Maximally entangled.

If *C*(*m*, *t*_*m*−1_, _*j*_ ⊕_*d*′_ 1) = 0 for any *m*, it is obvious that *t*_*mj*_ ≠ *t*_*m*′*j*_ for *m* ≠ *m*′.

If *C*(*m*,*t*_*m*−1_, _*j*_ ⊕_*d*′_ 1) = 1 for some *m* ≠ 0, from the definition of *t*_*mj*_ one has *t*_*m*−1_, _*j*_ ≠ *b*_*m*−1_. Note that *b*_*m*_ − *b*_*m*−1_ = 0 or 1, then *t*_*m*−1_, _*j*_ = *b*_*m*−1_ ⊕_*d*′_ 1.

From the definition of *t*_*mj*_, we also have *C*(*k* + 1, *t*_*kj*_ + 1) = 0 for *k* ≠ *m* − 1. Hence

5 $${t}_{kj}=\{\begin{array}{cc}{t}_{m-1,j}\,{\ominus }_{d^{\prime} }\,(m-1-k), & 0\,\leqslant \,k < m-1;\\ {t}_{mj}{\oplus }_{d^{\prime} }(k-m), & k\,\geqslant \,m.\end{array}$$

where $p\,\ominus {}_{d^{\prime} }m$ denotes (*p* − *m*) mod *d*′. In particular,

6 $${t}_{0j}={t}_{m-\mathrm{1,}j}\,\ominus {}_{d^{\prime} }\,(m-\mathrm{1),}\,\,\,\,{t}_{d-\mathrm{1,}j}={t}_{mj}{\oplus }_{d^{\prime} }(d-1-m\mathrm{).}$$

Then

7 $$\begin{array}{rcl}{t}_{d-\mathrm{1,}j}-{t}_{0j} & = & {t}_{mj}{\oplus }_{d^{\prime} }(d-1-m)-{t}_{m-\mathrm{1,}j}{\ominus }_{d^{\prime} }\,(m-\mathrm{1)}\\  & = & d-2+({t}_{mj}-{t}_{m-\mathrm{1,}j})\\  & = & d < d^{\prime} \mathrm{.}\end{array}$$

Hence *t*_*mj*_ ≠ *t*_*m*′*j*_ for *m* ≠ *m*′. Namely, the states |*ϕ*′_*j*,*n*_〉 in (3) are all maximally entangled.

(ii) Orthogonality.

We first show that |*t*_*mj*_〉 = |*t*_*mj*′_〉 if and only if *j* = *j*′.

Obviously, *t*_*mj*_ = *t*_*mj*′_ for *j* = *j*′. If *j* ≠ *j*′, without loss of generality, let $j^{\prime}  > j$. It is easy to show that *t*_*mj*_ ≠ *t*_*mj*′_ when *t*_*m*−1,*j*_ ≠ *t*_*m*−1,*j*′_. Otherwise, from the definition of *t*_*mj*_ we have ${t}_{mj}={t}_{m-\mathrm{1,}j^{\prime} }{\ominus }_{d^{\prime} }\,C(m,{t}_{m-\mathrm{1,}j}\oplus \mathrm{1)}$ when *C*(*m*, *t*_*m*−1,*j*_⊕_*d*′_1) = 1. Note that ${t}_{m-\mathrm{1,}j^{\prime} }={b}_{m-1}{\ominus }_{d^{\prime} }1$ when *C*(*m*, *t*_*m*−1_,_,*j*_⊕_*d*′_1) = 1, as proved in (i). Therefore, *t*_*m*−1_, _*j*′_ = *b*_*m*−1_, which contradicts to the definition of *t*_*mj*_. Furthermore, *t*_*mj*_ ≠ *t*_*mj*′_ when *t*_0*j*_ ≠ *t*_0*j*′_. Therefore,

8 $$\begin{array}{rcl}\langle {\varphi }{^{\prime} }_{j,n}|{\varphi }{^{\prime} }_{j^{\prime} ,n^{\prime} }\rangle  & = & \frac{1}{d}\sum _{m\mathrm{=0}}^{d-1}\overline{{\omega }^{n^{\prime} m}}{\omega }^{nm}\langle {t}_{mj}|{t}_{mj^{\prime} }\rangle \\  & = & \frac{1}{d}\sum _{m=0}^{d-1}{\omega }^{(n-n^{\prime} )m}{\delta }_{jj^{\prime} }\\  & = & {\delta }_{nn^{\prime} }{\delta }_{jj^{\prime} }\mathrm{.}\end{array}$$

Thus, the *d*(*d*′ − 1) states {|*ϕ*_*j*, *n*_〉} in (3) constitutes an MEB in *V*. Furthermore, there exist no MEBs in *V*^⊥^ because *N* < *d*. Hence {|*ϕ*_*j*, *n*_〉} is a UMEB in *M*_*d*×*d*′_, as well as in ${{\mathbb{C}}}^{d}\otimes {{\mathbb{C}}}^{d^{\prime} }$.

### **Example 1**.

Constructing two UMEBs in *C*^5^ ⊗ *C*^6^, where as $$V=(\begin{array}{cccccc}0 & 0 & 0 & \ast  & 0 & 0\\ 0 & \ast  & 0 & 0 & 0 & 0\\ 0 & 0 & 0 & \ast  & 0 & 0\\ 0 & 0 & 0 & 0 & 0 & \ast \\ 0 & 0 & 0 & \ast  & 0 & 0\end{array}).$$

We can get the following matrix *V*′ by using suitable unitary transformation on *V*,

$$V^{\prime} =PVQ=(\begin{array}{cccccc}\ast  & 0 & 0 & 0 & 0 & 0\\ \ast  & 0 & 0 & 0 & 0 & 0\\ \ast  & 0 & 0 & 0 & 0 & 0\\ 0 & \ast  & 0 & 0 & 0 & 0\\ 0 & 0 & \ast  & 0 & 0 & 0\end{array}),$$

where

$$P=(\begin{array}{ccccc}1 & 0 & 0 & 0 & 0\\ 0 & 0 & 1 & 0 & 0\\ 0 & 0 & 0 & 0 & 1\\ 0 & 1 & 0 & 0 & 0\\ 0 & 0 & 0 & 1 & 0\end{array}),\,\,Q=(\begin{array}{cccccc}0 & 0 & 0 & 1 & 0 & 0\\ 0 & 1 & 0 & 0 & 0 & 0\\ 0 & 0 & 0 & 0 & 1 & 0\\ 1 & 0 & 0 & 0 & 0 & 0\\ 0 & 0 & 0 & 0 & 0 & 1\\ 0 & 0 & 1 & 0 & 0 & 0\end{array}).$$

According to Theorem 1, we first construct an MEB $\{|{\varphi }{^{\prime} }_{j}\rangle {\}}_{j=1}^{25}$ in *V*′, i.e. a UMEB in *C*^5^ ⊗ *C*^6^ as follows:

9 $$\{\begin{array}{ccc}|\varphi {^{\prime} }_{1,2,3,4,5}\rangle  & = & \frac{1}{\sqrt{5}}(|01^{\prime} \rangle +\alpha |12^{\prime} \rangle +{\alpha }^{2}|23^{\prime} \rangle +{\alpha }^{3}|34^{\prime} \rangle +{\alpha }^{4}|45^{\prime} \rangle ),\\ |\varphi {^{\prime} }_{6,7,8,9,10}\rangle  & = & \frac{1}{\sqrt{5}}(|02^{\prime} \rangle +\alpha |13^{\prime} \rangle +{\alpha }^{2}|24^{\prime} \rangle +{\alpha }^{3}|35^{\prime} \rangle +{\alpha }^{4}|40{\rm{^{\prime} }}\rangle ),\\ |\varphi {^{\prime} }_{11,12,13,14,15}\rangle  & = & \frac{1}{\sqrt{5}}(|03^{\prime} \rangle +\alpha |14^{\prime} \rangle +{\alpha }^{2}|25^{\prime} \rangle +{\alpha }^{3}|30^{\prime} \rangle +{\alpha }^{4}|41^{\prime} \rangle ),\\ |\varphi {^{\prime} }_{16,17,18,19,20}\rangle  & = & \frac{1}{\sqrt{5}}(|04^{\prime} \rangle +\alpha |15^{\prime} \rangle +{\alpha }^{2}|2{1}^{^{\prime} }\rangle +{\alpha }^{3}|32^{\prime} \rangle +{\alpha }^{4}|43^{\prime} \rangle ),\\ |\varphi {^{\prime} }_{21,22,23,24,25}\rangle  & = & \frac{1}{\sqrt{5}}(|05^{\prime} \rangle +\alpha |11^{\prime} \rangle +{\alpha }^{2}|22^{\prime} \rangle +{\alpha }^{3}|33^{\prime} \rangle +{\alpha }^{4}|44^{\prime} \rangle ),\end{array}$$

where $$\alpha =\mathrm{1,}\,{\omega }_{5},\,{\omega }_{5}^{2},\,{\omega }_{5}^{3},\,{\omega }_{5}^{4}$$.

By inverse unitary transformation |*ϕ*_*j*_〉 = (*P*^−1^ ⊗ *Q*^−1^)|*ϕ*_*j*_′〉, we get the following MEB $\{|{{\varphi }}_{j}\rangle {\}}_{j=1}^{25}$ in *V*, i.e., another UMEB in *C*^5^ ⊗ *C*^6^:

10 $$\{\begin{array}{ccc}|{\varphi }_{1,2,3,4,5}\rangle  & = & \frac{1}{\sqrt{5}}(|01^{\prime} \rangle +\alpha |25^{\prime} \rangle +{\alpha }^{2}|40^{\prime} \rangle +{\alpha }^{3}|12^{\prime} \rangle +{\alpha }^{4}|34^{\prime} \rangle ),\\ |{\varphi }_{6,7,8,9,10}\rangle  & = & \frac{1}{\sqrt{5}}(|05^{\prime} \rangle +\alpha |20^{\prime} \rangle +{\alpha }^{2}|42^{\prime} \rangle +{\alpha }^{3}|14^{\prime} \rangle +{\alpha }^{4}|33^{\prime} \rangle ),\\ |{\varphi }_{11,12,13,14,15}\rangle  & = & \frac{1}{\sqrt{5}}(|00^{\prime} \rangle +\alpha |22^{\prime} \rangle +{\alpha }^{2}|44^{\prime} \rangle +{\alpha }^{3}|13^{\prime} \rangle +{\alpha }^{4}|31^{\prime} \rangle ),\\ |{\varphi }_{16,17,18,19,20}\rangle  & = & \frac{1}{\sqrt{5}}(|02^{\prime} \rangle +\alpha |24^{\prime} \rangle +{\alpha }^{2}|41^{\prime} \rangle +{\alpha }^{3}|15^{\prime} \rangle +{\alpha }^{4}|30^{\prime} \rangle ),\\ |{\varphi }_{21,22,23,24,25}\rangle  & = & \frac{1}{\sqrt{5}}(|04^{\prime} \rangle +\alpha |21^{\prime} \rangle +{\alpha }^{2}|45^{\prime} \rangle +{\alpha }^{3}|10^{\prime} \rangle +{\alpha }^{4}|32^{\prime} \rangle ),\end{array}$$

where $$\alpha =\mathrm{1,}\,{\omega }_{5},\,{\omega }_{5}^{2},\,{\omega }_{5}^{3},\,{\omega }_{5}^{4}$$.

### **Remark 1**.

Actually both (9) and (10) are UMEBs in ${{\mathbb{C}}}^{5}\otimes {{\mathbb{C}}}^{6}$. However, they are different although they can be unitarily transformed to each other. We will reveal the difference in the following example.

### **Example 2**.

Constructing a UMEB in ${{\mathbb{C}}}^{5}\otimes {{\mathbb{C}}}^{12}$, whereas

11 $$V=({V}_{1}|{V}_{2})=(\begin{array}{cccccccccccc}0 & 0 & 0 & \ast  & 0 & 0 & \ast  & 0 & 0 & 0 & 0 & 0\\ 0 & 0 & 0 & \ast  & 0 & 0 & 0 & 0 & 0 & \ast  & 0 & 0\\ 0 & \ast  & 0 & 0 & 0 & 0 & \ast  & 0 & 0 & 0 & 0 & 0\\ 0 & 0 & 0 & \ast  & 0 & 0 & 0 & 0 & 0 & \ast  & 0 & 0\\ 0 & 0 & 0 & \ast  & 0 & 0 & \ast  & 0 & 0 & 0 & 0 & 0\end{array}).$$

One can easily get the following simple formations ${V^{\prime} }_{1}$ and ${V^{\prime} }_{2}$ from *V*_1_ and *V*_2_ by elementary transformation respectively:

12 $${V}_{1}^{\prime} ={P}_{1}{V}_{1}{Q}_{1}=(\begin{array}{cccccc}\ast  & 0 & 0 & 0 & 0 & 0\\ 0 & \ast  & 0 & 0 & 0 & 0\\ 0 & \ast  & 0 & 0 & 0 & 0\\ 0 & \ast  & 0 & 0 & 0 & 0\\ 0 & \ast  & 0 & 0 & 0 & 0\end{array}),\,\,\,{V^{\prime} }_{2}={P}_{2}{V}_{2}{Q}_{2}=(\begin{array}{cccccc}\ast  & 0 & 0 & 0 & 0 & 0\\ \ast  & 0 & 0 & 0 & 0 & 0\\ \ast  & 0 & 0 & 0 & 0 & 0\\ 0 & \ast  & 0 & 0 & 0 & 0\\ 0 & \ast  & 0 & 0 & 0 & 0\end{array}).$$

Then following Theorem 1 we can construct the following UMEBs $\{|{{\varphi }}_{j^{\prime} }\rangle {\}}_{j=1}^{25}$ and $\{|{\psi }_{j^{\prime} }\rangle {\}}_{j\mathrm{=1}}^{25}$ in ${V^{\prime} }_{1}$ and ${V^{\prime} }_{2}$ respectively:

13 $$\{\begin{array}{cc}|\varphi {^{\prime} }_{1,2,3,4,5}\rangle  & =\,\,\frac{1}{\sqrt{5}}(|01^{\prime} \rangle +\alpha |12^{\prime} \rangle +{\alpha }^{2}|23^{\prime} \rangle +{\alpha }^{3}|34^{\prime} \rangle +{\alpha }^{4}|45^{\prime} \rangle ),\\ |\varphi {^{\prime} }_{6,7,8,9,10}^{\prime} \rangle  & =\,\,\frac{1}{\sqrt{5}}(|02^{\prime} \rangle +\alpha |13^{\prime} \rangle +{\alpha }^{2}|24^{\prime} \rangle +{\alpha }^{3}|35^{\prime} \rangle +{\alpha }^{4}|40^{\prime} \rangle ),\\ |\varphi {^{\prime} }_{11,12,13,14,15}^{\prime} \rangle  & =\,\,\frac{1}{\sqrt{5}}(|03^{\prime} \rangle +\alpha |14^{\prime} \rangle +{\alpha }^{2}|25^{\prime} \rangle +{\alpha }^{3}|30^{\prime} \rangle +{\alpha }^{4}|42^{\prime} \rangle ),\\ |\varphi {^{\prime} }_{16,17,18,19,20}^{\prime} \rangle  & =\,\,\frac{1}{\sqrt{5}}(|04^{\prime} \rangle +\alpha |15^{\prime} \rangle +{\alpha }^{2}|20^{\prime} \rangle +{\alpha }^{3}|32^{\prime} \rangle +{\alpha }^{4}|43^{\prime} \rangle ),\\ |\varphi {^{\prime} }_{21,22,23,24,25}^{\prime} \rangle  & =\,\,\frac{1}{\sqrt{5}}(|05^{\prime} \rangle +\alpha |10^{\prime} \rangle +{\alpha }^{2}|22^{\prime} \rangle +{\alpha }^{3}|33^{\prime} \rangle +{\alpha }^{4}|44^{\prime} \rangle ),\end{array}$$

14 $$\{\begin{array}{cc}|\psi {^{\prime} }_{1,2,3,4,5}\rangle  & =\,\,\frac{1}{\sqrt{5}}(|07^{\prime} \rangle +\alpha |18^{\prime} \rangle +{\alpha }^{2}|29^{\prime} \rangle +{\alpha }^{3}|3,10^{\prime} \rangle +{\alpha }^{4}|4,11^{\prime} \rangle ),\\ |\psi {^{\prime} }_{6,7,8,9,10}\rangle  & =\,\,\frac{1}{\sqrt{5}}(|08^{\prime} \rangle +\alpha |19^{\prime} \rangle +{\alpha }^{2}|2,10^{\prime} \rangle +{\alpha }^{3}|3,11^{\prime} \rangle +{\alpha }^{4}|46^{\prime} \rangle ),\\ |\psi {^{\prime} }_{11,12,13,14,15}\rangle  & =\,\,\frac{1}{\sqrt{5}}(|09^{\prime} \rangle +\alpha |1,10^{\prime} \rangle +{\alpha }^{2}|2,11^{\prime} \rangle +{\alpha }^{3}|3,\,6^{\prime} \rangle +{\alpha }^{4}|48^{\prime} \rangle ),\\ |\psi {^{\prime} }_{16,17,18,19,20}\rangle  & =\,\,\frac{1}{\sqrt{5}}(|0,\,10^{\prime} \rangle +\alpha |1,11^{\prime} \rangle +{\alpha }^{2}|27^{\prime} \rangle +{\alpha }^{3}|38^{\prime} \rangle +{\alpha }^{4}|49^{\prime} \rangle ),\\ |\psi {^{\prime} }_{21,22,23,24,25}\rangle  & =\,\,\frac{1}{\sqrt{5}}(|0,\,11^{\prime} \rangle +\alpha |17^{\prime} \rangle +{\alpha }^{2}|28^{\prime} \rangle +{\alpha }^{3}|39^{\prime} \rangle +{\alpha }^{4}|4,\,10^{\prime} \rangle )\end{array}$$

where $$\alpha =\mathrm{1,}\,{\omega }_{5},\,{\omega }_{5}^{2},\,{\omega }_{5}^{3},\,{\omega }_{5}^{4}$$.

By inverse transformation $|{{\varphi }}_{j}\rangle =({P}_{1}^{-1}\otimes {Q}_{1}^{-1})|{\varphi }{^{\prime} }_{j}\rangle$ and $|{\psi }_{j}\rangle =({P}_{2}^{-1}\otimes {Q}_{2}^{-1})|\psi {^{\prime} }_{j}\rangle$, we can obtain the following UMEBs $\{|{{\varphi }}_{j}\rangle {\}}_{j\mathrm{=1}}^{25}$ and $\{|{\psi }_{j}\rangle {\}}_{j\mathrm{=1}}^{25}$ in *V*_1_ and *V*_2_, respectively,

15 $$\{\begin{array}{ccc}|{\varphi }_{1,2,3,4,5}\rangle  & = & \frac{1}{\sqrt{5}}(|01^{\prime} \rangle +\alpha |12^{\prime} \rangle +{\alpha }^{2}|23^{\prime} \rangle +{\alpha }^{3}|34^{\prime} \rangle +{\alpha }^{4}|45^{\prime} \rangle ),\\ |{\varphi }_{6,7,8,9,10}\rangle  & = & \frac{1}{\sqrt{5}}(|02^{\prime} \rangle +\alpha |13^{\prime} \rangle +{\alpha }^{2}|24^{\prime} \rangle +{\alpha }^{3}|35^{\prime} \rangle +{\alpha }^{4}|40^{\prime} \rangle ),\\ |{\varphi }_{11,12,13,14,15}\rangle  & = & \frac{1}{\sqrt{5}}(|03^{\prime} \rangle +\alpha |14^{\prime} \rangle +{\alpha }^{2}|25^{\prime} \rangle +{\alpha }^{3}|30^{\prime} \rangle +{\alpha }^{4}|42^{\prime} \rangle ),\\ |{\varphi }_{16,17,18,19,20}\rangle  & = & \frac{1}{\sqrt{5}}(|04^{\prime} \rangle +\alpha |15^{\prime} \rangle +{\alpha }^{2}|20^{\prime} \rangle +{\alpha }^{3}|32^{\prime} \rangle +{\alpha }^{4}|43^{\prime} \rangle ),\\ |{\varphi }_{21,22,23,24,25}\rangle  & = & \frac{1}{\sqrt{5}}(|05^{\prime} \rangle +\alpha |10^{\prime} \rangle +{\alpha }^{2}|22^{\prime} \rangle +{\alpha }^{3}|33^{\prime} \rangle +{\alpha }^{4}|44^{\prime} \rangle ),\end{array}$$

16 $$\{\begin{array}{ccc}|{\psi }_{1,2,3,4,5}\rangle  & = & \frac{1}{\sqrt{5}}(|07^{\prime} \rangle +\alpha |18^{\prime} \rangle +{\alpha }^{2}|29^{\prime} \rangle +{\alpha }^{3}|3,10^{\prime} \rangle +{\alpha }^{4}|4,11^{\prime} \rangle ),\\ |{\psi }_{6,7,8,9,10}\rangle  & = & \frac{1}{\sqrt{5}}(|08^{\prime} \rangle +\alpha |19^{\prime} \rangle +{\alpha }^{2}|2,10^{\prime} \rangle +{\alpha }^{3}|3,11^{\prime} \rangle +{\alpha }^{4}|46^{\prime} \rangle ),\\ |{\psi }_{11,12,13,14,15}\rangle  & = & \frac{1}{\sqrt{5}}(|09^{\prime} \rangle +\alpha |1,10^{\prime} \rangle +{\alpha }^{2}|2,11^{\prime} \rangle +{\alpha }^{3}|36^{\prime} \rangle +{\alpha }^{4}|48^{\prime} \rangle ),\\ |{\psi }_{16,17,18,19,20}\rangle  & = & \frac{1}{\sqrt{5}}(|0,10^{\prime} \rangle +\alpha |1,11^{\prime} \rangle +{\alpha }^{2}|27^{\prime} \rangle +{\alpha }^{3}|38^{\prime} \rangle +{\alpha }^{4}|49^{\prime} \rangle ),\\ |{\psi }_{21,22,23,24,25}\rangle  & = & \frac{1}{\sqrt{5}}(|0,11^{\prime} \rangle +\alpha |17^{\prime} \rangle +{\alpha }^{2}|28^{\prime} \rangle +{\alpha }^{3}|39^{\prime} \rangle +{\alpha }^{4}|4,10^{\prime} \rangle ).\end{array}$$

Thus, {|*ϕ*_*j*_〉} ∪ {|*ψ*_*j*_〉} constitutes a UMEB in ${{\mathbb{C}}}^{5}\otimes {{\mathbb{C}}}^{12}$ with *V* in (8). However, neither $({P}_{1}^{-1}\otimes {Q}_{1}^{-1})$ nor $({P}_{2}^{-1}\otimes {Q}_{2}^{-1})$ can transform $\{|{\psi ^{\prime} }_{j}\rangle \}\cup \{|{\varphi ^{\prime} }_{j}\rangle \}$ to $\{|{\varphi }_{j}\rangle \}\cup \{|{\psi }_{j}\rangle \}$, which shows the difference between (9) and (10).

## *d(d′ − r)*-member UMEBs in ${{\mathbb{C}}}^{d}\otimes {{\mathbb{C}}}^{d^{\prime} }$

In this section, we construct UMEBs consisting of fewer elements in ${{\mathbb{C}}}^{d}\otimes {{\mathbb{C}}}^{d^{\prime} }$. The following theorem provides a systematic way of constructing *d*(*d*′ − *r*)-member UMEBs in ${{\mathbb{C}}}^{d}\otimes {{\mathbb{C}}}^{d^{\prime} }$, *r* = 1, 2, …, *d* − 1, that is to say, it presents *d* − 1 constructions of UMEB in ${{\mathbb{C}}}^{d}\otimes {{\mathbb{C}}}^{d^{\prime} }$.

### **Theorem 2**.

Let $d^{\prime} ={\sum }_{i\mathrm{=1}}^{s}{a}_{i}+r$, where $s\,\geqslant \,\mathrm{1,}\,{a}_{i}\,\geqslant \,d,\,0 < r < d$. Then the following vectors constitute a *d*(*d*′ − *r*)-member UMEB in *C*^*d*^ ⊗ *C*^*d*′^:

17 $$|{{\varphi }}_{l,j,n}\rangle =\frac{1}{\sqrt{d}}\sum _{m\mathrm{=0}}^{d-1}{\omega }_{d}^{nm}|m\rangle |{b}_{j}+({l}_{j+i}{\oplus }_{{a}_{j+1}}m)\rangle ,{}_{j+1}=0,\,\mathrm{1,}\,\cdots ,{a}_{j+1}-\mathrm{1,}$$

where $${b}_{j}={\sum }_{k\mathrm{=1}}^{j}{a}_{k}$$; *j* = 0, 1,…, *s* − 1; *n* = 0, 1 …, *d* − 1.

### Proof.

(i) It is obvious that |*ϕ*_*l*,*j*,*n*_〉 in (16) are all maximally entangled.

(ii) Orthogonality,

18 $$\begin{array}{ll}\langle {{\varphi }}_{l,j,n}|{{\varphi }}_{l^{\prime} ,j^{\prime} ,n^{\prime} }\rangle  & =\,\,\frac{1}{d}\sum _{m\mathrm{=0}}^{d-1}\overline{{\omega }_{d}^{n^{\prime} m}}{\omega }_{d}^{nm}\langle {b}_{j}+({l}_{j+1}{\oplus }_{{a}_{j+1}}m)|{b}_{j^{\prime} }+(l{^{\prime} }_{j^{\prime} +1}{\oplus }_{{a}_{j^{\prime} +1}}m)\rangle \\  & =\,\,\frac{1}{d}\sum _{m\mathrm{=0}}^{d-1}{\omega }_{d}^{(n-n^{\prime} )m}{\delta }_{jj^{\prime} }\langle {l}_{j+1}{\oplus }_{{a}_{j+1}}m|l{^{\prime} }_{j^{\prime} +1}{\oplus }_{{a}_{j^{\prime} +1}}m\rangle \\  & =\,\,\frac{1}{d}\sum _{m\mathrm{=0}}^{d-1}{\omega }_{d}^{(n-n^{\prime} )m}{\delta }_{jj^{\prime} }{\delta }_{ll^{\prime} }\\  & =\,\,{\delta }_{nn^{\prime} }{\delta }_{jj^{\prime} }{\delta }_{ll}\mathrm{.}\end{array}$$

(iii) Denote *M*_1_ the *d* ⊗ (*d*′ − *n*) matrix space, a subspace of *M*_*d*×*d*′_. Since the number of {|*ϕ*_*l*,*j*,*n*_〉} in (17) equals to the dimension of *M*_1_, {|*ϕ*_*l*,*j*,*n*_〉} is an MEB of *M*_1_. Moreover, since ${M}_{1}^{\perp }$ is a *d* × *r* matrix space and *r* < *d*, there contains no UMEB in ${M}_{1}^{\perp }$. From Lemma 1, {|*ϕ*_*l*,*j*,*n*_〉} is a UMEB of ${{\mathbb{C}}}^{d}\otimes {{\mathbb{C}}}^{d^{\prime} }$.

### **Example 3**.

UMEBs in $${{\mathbb{C}}}^{3}\otimes {{\mathbb{C}}}^{10}$$.

Obviously, 10 = 4 + 5 + 1 or 10 = 4 + 4 + 2. According to Theorem 2, we can construct the following 27-number UMEB (19) and 24-number UMEB (20) in ${{\mathbb{C}}}^{3}\otimes {{\mathbb{C}}}^{10}$ respectively.

19 $$\{\begin{array}{ccc}|{\varphi }_{1,2,3}\rangle  & = & \frac{1}{\sqrt{3}}(|00^{\prime} \rangle +\alpha |11^{\prime} \rangle +{\alpha }^{2}|22^{\prime} \rangle ),\\ |{\varphi }_{4,5,6}\rangle  & = & \frac{1}{\sqrt{3}}(|01^{\prime} \rangle +\alpha |12^{\prime} \rangle +{\alpha }^{2}|23^{\prime} \rangle ),\\ |{\varphi }_{7,8,9}\rangle  & = & \frac{1}{\sqrt{3}}(|02^{\prime} \rangle +\alpha |13^{\prime} \rangle +\alpha 2|20^{\prime} \rangle ),\\ |{\varphi }_{10,11,12}\rangle  & = & \frac{1}{\sqrt{3}}(|03^{\prime} \rangle +\alpha |10^{\prime} \rangle +{\alpha }^{2}|21^{\prime} \rangle ),\\ |{\varphi }_{13,14,15}\rangle  & = & \frac{1}{\sqrt{3}}(|04^{\prime} \rangle +\alpha |15^{\prime} \rangle +{\alpha }^{2}|26^{\prime} \rangle ),\\ |{\varphi }_{16,17,18}\rangle  & = & \frac{1}{\sqrt{3}}(|05^{\prime} \rangle +\alpha |16^{\prime} \rangle +{\alpha }^{2}|27^{\prime} \rangle ),\\ |{\varphi }_{19,20,21}\rangle  & = & \frac{1}{\sqrt{3}}(|06^{\prime} \rangle +\alpha |17^{\prime} \rangle +{\alpha }^{2}|28^{\prime} \rangle ),\\ |{\varphi }_{22,23,24}\rangle  & = & \frac{1}{\sqrt{3}}(|07^{\prime} \rangle +\alpha |18^{\prime} \rangle +{\alpha }^{2}|24^{\prime} \rangle ),\\ |{\varphi }_{25,26,27}\rangle  & = & \frac{1}{\sqrt{3}}(|08^{\prime} \rangle +\alpha |14^{\prime} \rangle +{\alpha }^{2}|25^{\prime} \rangle ),\end{array}$$

and

20 $$\{\begin{array}{ccc}|{\varphi }_{1,2,3}\rangle  & = & \frac{1}{\sqrt{3}}(|00^{\prime} \rangle +\alpha |11^{\prime} \rangle +{\alpha }^{2}|22^{\prime} \rangle ),\\ |{\varphi }_{4,5,6}\rangle  & = & \frac{1}{\sqrt{3}}(|01^{\prime} \rangle +\alpha |12^{\prime} \rangle +{\alpha }^{2}|23^{\prime} \rangle ),\\ |{\varphi }_{7,8,9}\rangle  & = & \frac{1}{\sqrt{3}}(|02^{\prime} \rangle +\alpha |13^{\prime} \rangle +{\alpha }^{2}|20^{\prime} \rangle ),\\ |{\varphi }_{10,11,12}\rangle  & = & \frac{1}{\sqrt{3}}(|03^{\prime} \rangle +\alpha |10^{\prime} \rangle +{\alpha }^{2}|21^{\prime} \rangle ),\\ |{\varphi }_{13,14,15}\rangle  & = & \frac{1}{\sqrt{3}}(|04^{\prime} \rangle +\alpha |15^{\prime} \rangle +{\alpha }^{2}|26^{\prime} \rangle ),\\ |{\varphi }_{16,17,18}\rangle  & = & \frac{1}{\sqrt{3}}(|05^{\prime} \rangle +\alpha |16^{\prime} \rangle +{\alpha }^{2}|27^{\prime} \rangle ),\\ |{\varphi }_{19,20,21}\rangle  & = & \frac{1}{\sqrt{3}}(|06^{\prime} \rangle +\alpha |17^{\prime} \rangle +{\alpha }^{2}|24^{\prime} \rangle ),\\ |{\varphi }_{22,23,24}\rangle  & = & \frac{1}{\sqrt{3}}(|07^{\prime} \rangle +\alpha |14^{\prime} \rangle +{\alpha }^{2}|25^{\prime} \rangle ),\end{array}$$

where $$\alpha =\mathrm{1,}\,{\omega }_{3},\,{\omega }_{3}^{2}$$.

### **Remark 2**.

Theorem 2 gives a very large number of UMEBs in *C*^*d*^ ⊗ *C*^*d*′^, which is more than all the previous numbers. For example, the 27-number UMEB (19) and 24-number UMEB (20) in Example 3 are only two kinds of UMEBs in ${{\mathbb{C}}}^{3}\otimes {{\mathbb{C}}}^{10}$. Actually according to Theorem 2, there are five more kinds of UMEBs in ${{\mathbb{C}}}^{3}\otimes {{\mathbb{C}}}^{10}$, since 10 = 3 + 5 + 2, 10 = 3 + 6 + 1, 10 = 3 + 3 + 3 + 1, 10 = 8 + 2 and 10 = 9 + 1.

### **Remark 3**.

Theorem 2 in ref.^21^ is a special case of the above Theorem 2 at *d*′ = *a*_1_ + *r*. Theorem 1 in ref.^20^ and Theorem 1 in ref.^21^ are both special cases of our Theorem 1, where all the *a*_*i*_ are equal.

## Conclusion

We have provided new constructions of unextendible maximally entangled bases in arbitrary bipartite spaces ${{\mathbb{C}}}^{d}\otimes {{\mathbb{C}}}^{d^{\prime} }$. We have presented a systematic way of constructing *d*(*d*′ − 1)-member UMEB in ${{\mathbb{C}}}^{d}\otimes {{\mathbb{C}}}^{d^{\prime} }$, and constructed two different UMEBs in ${{\mathbb{C}}}^{3}\otimes {{\mathbb{C}}}^{6}$ and ${{\mathbb{C}}}^{3}\otimes {{\mathbb{C}}}^{12}$ respectively. We have established a flexible method to construct *d*(*d* − *r*)-number UMEBs in ${{\mathbb{C}}}^{d}\otimes {{\mathbb{C}}}^{d^{\prime} }$, *r* = 1, 2, …, *d* − 1. Namely, we have presented more than *d* − 1 constructions of UMEBs in ${{\mathbb{C}}}^{d}\otimes {{\mathbb{C}}}^{d^{\prime} }$. Such generalized the main results in ref.^21^ and ref.^20^. We have also shown 27-number UMEB and 24-number UMEB in ${{\mathbb{C}}}^{3}\otimes {{\mathbb{C}}}^{10}$, respectively.
